# A preterm neonate with infantile liver failure syndrome 1 due to leucyl‐tRNA synthetase 1 gene (*LARS1*) mutations with a histopathologic phenotype of neonatal hemochromatosis

**DOI:** 10.1002/jpr3.70203

**Published:** 2026-07-29

**Authors:** Adrienne Bruder, Naomi Laventhal, Raja Rabah, Jacqueline Meadow

**Affiliations:** ^1^ Department of Pediatrics, Neonatal‐Perinatal Medicine University of Michigan Ann Arbor Michigan USA; ^2^ Department of Pathology, Pediatric and Perinatal Pathology University of Michigan Ann Arbor Michigan USA

**Keywords:** fulminant liver failure, intrauterine growth restriction, neonatal liver failure

## Abstract

We report a case of a premature, growth‐restricted female infant with feeding intolerance and coagulopathy, treated initially for sepsis, who progressed to neonatal acute liver failure and end‐stage hepatic encephalopathy after a prolonged hospitalization with extensive diagnostic evaluation, and was found by autopsy to have histopathologic evidence of neonatal hemochromatosis in addition to a rare autosomal recessive disorder of infantile liver failure syndrome type 1.

## 1 INTRODUCTION

Common pathologies in premature infants with severe intrauterine growth restriction (IUGR), such as sepsis and necrotizing enterocolitis, have overlapping clinical findings with primary liver failure syndromes, making liver failure syndromes hard to recognize.

## 2 CASE REPORT

A female infant was born at 32 weeks gestation by cesarean section to a G1P0 mother for maternal preeclampsia and non‐reassuring fetal heart tones. The pregnancy was complicated by IUGR and dilated bowel without ascites. Apgar scores were 5, 7, and 8 at 1, 5, and 10 min, respectively. There was green amniotic fluid. She was small for gestational age (weight 920 g, second percentile; length 37 cm, fifth percentile; head circumference 26 cm, third percentile). Immediate neonatal course included respiratory failure requiring intubation and surfactant administration, and congenital anemia (hemoglobin 10.7 g/dL).

The patient spontaneously passed small pellet‐like stools, which transitioned to normal‐appearing meconium. Trophic breastmilk feedings were initiated but stopped due to abdominal distention. A contrast enema revealed no obstruction or malrotation based on normal cecum position, with small diameter of the large intestine noted. During the next 2 weeks, the patient had continued anemia and received four packed red blood cell (PRBC) transfusions, prompting sepsis evaluation (7 days of gentamicin and piperacillin‐tazobactam, negative blood cultures). Due to persistent leukocytosis, she was again evaluated for sepsis and treated with meropenem, vancomycin, and micafungin. Her initial liver enzymes on day of life one were normal (aspartate transaminase [AST] 40 U/L [0–60 U/L], alanine aminotransferase [ALT] 6 U/L [0–40 U/L], alkaline phosphatase 246 U/L [83–248 U/L], albumin 2.3 g/dL [2.2–4.0 g/dL]), and total and direct bilirubin were as expected for a newborn (6.1/0.4 mg/dL). Coagulation factors were not obtained at birth.

At 3 weeks of life, she remained intubated with anasarca and had new‐onset abdominal distention, thrombocytopenia, and hypoalbuminemia. Coagulation factors were drawn for the first time in her life, demonstrating severe coagulopathy (international normalized ratio [INR] 3.84, prothrombin time [PT] 40.2 s [12–14.5 s], prothrombin time test [PTT] 97.0 s [22.5–35 s], D‐Dimer 3000 mg/L [< 500 mg/L], fibrinogen <60 mg/dL [150–470 mg/dL]). She was transferred to our Level IV neonatal intensive care unit (NICU) with presumed sepsis from abdominal perforation. Her antimicrobial regimen was changed to meropenem and fluconazole for a total of 15 days. Abdominal ultrasound revealed nonspecific echogenic hepatic lesions and an extrahepatic portosystemic shunt with hepato‐fugal flow within the right and left portal veins and normal flow within the main portal vein. A paracentesis yielded 125 mL of ascites, which did not grow on aerobic, anaerobic, or fungal culture (though it was pre‐treated). Diagnostic evaluations for infectious hepatitis, including herpes simplex virus, cytomegalovirus, and enterovirus, were negative.

Although the age of presentation was not typical for gestational alloimmune liver disease (GALD), the combination of jaundice, edema, coagulopathy, and hypoalbuminemia without significant liver enzyme elevation prompted further investigation for GALD. Salivary gland biopsy resulted negative for iron staining by histopathology. At 4 weeks of life, after prolonged antibiotic treatment without improvement, intravenous immunoglobulin (IVIG) was given empirically for treatment of GALD. Liver enzymes were repeatedly normal (AST 31–62 U/L [≤70 U/L], ALT 13–25 U/L [10–49 U/L]). Initial ammonia (157 mmol/L [11–60 mmol/L]) and ferritin (383.2 ng/mL [7.0–271 ng/mL]) were mildly elevated, and α‐fetoprotein was 35,584 ng/mL. She had persistent anemia despite eight PRBC transfusions (mixed picture for hemolysis with low reticulocyte count (0.41% [0.63%–1.39%] and mildly elevated lactate dehydrogenase [343 U/L] but peak bilirubin of 35.6/11.4 mg/dL), and hypoalbuminemia despite multiple doses of albumin and protein intake of 3.5 g/kg/day). She had significant coagulopathy refractory to nearly daily fresh frozen plasma and cryoprecipitate transfusions (INR 1.8–5.9 [0.81–1.3], PT 18.2–55.2 s [11.3–16.2 s], PTT 55.3–136.1 s [27–52 s], fibrinogen 76–256 mg/dL [170–510 mg/dL]).

She developed worsening respiratory failure, hypotension, renal failure, and profound anasarca. At 7 weeks of age, she was again treated empirically for GALD with a second dose of IVIG and double volume exchange transfusion. Unfortunately, her liver failure progressed. She developed hepatic encephalopathy and seizures and died at 57 days of age after transition to comfort‐focused care.

**Figure 1:**
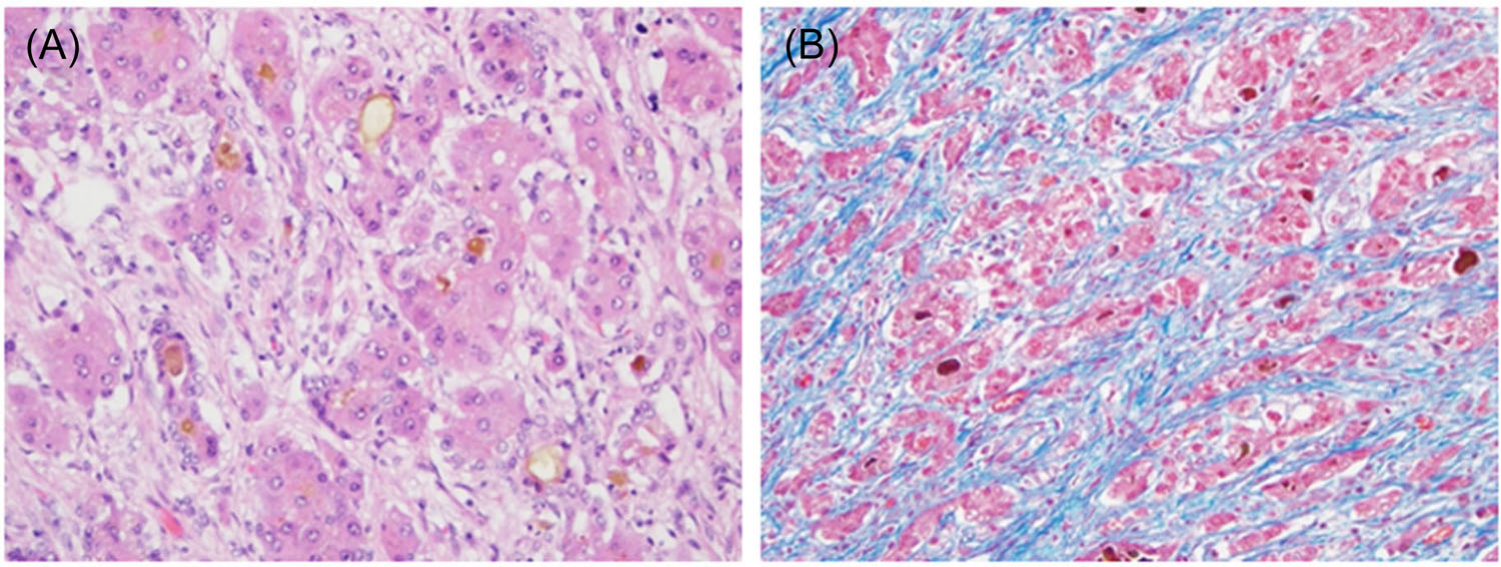
The hepatic parenchyma is replaced by giant‐cell or pseudoacinar transformation, fibrosis, and canalicular bile plugs (A, ×400). Trichrome stain highlights diffuse fibrosis (B, ×400).

**Figure 2:**
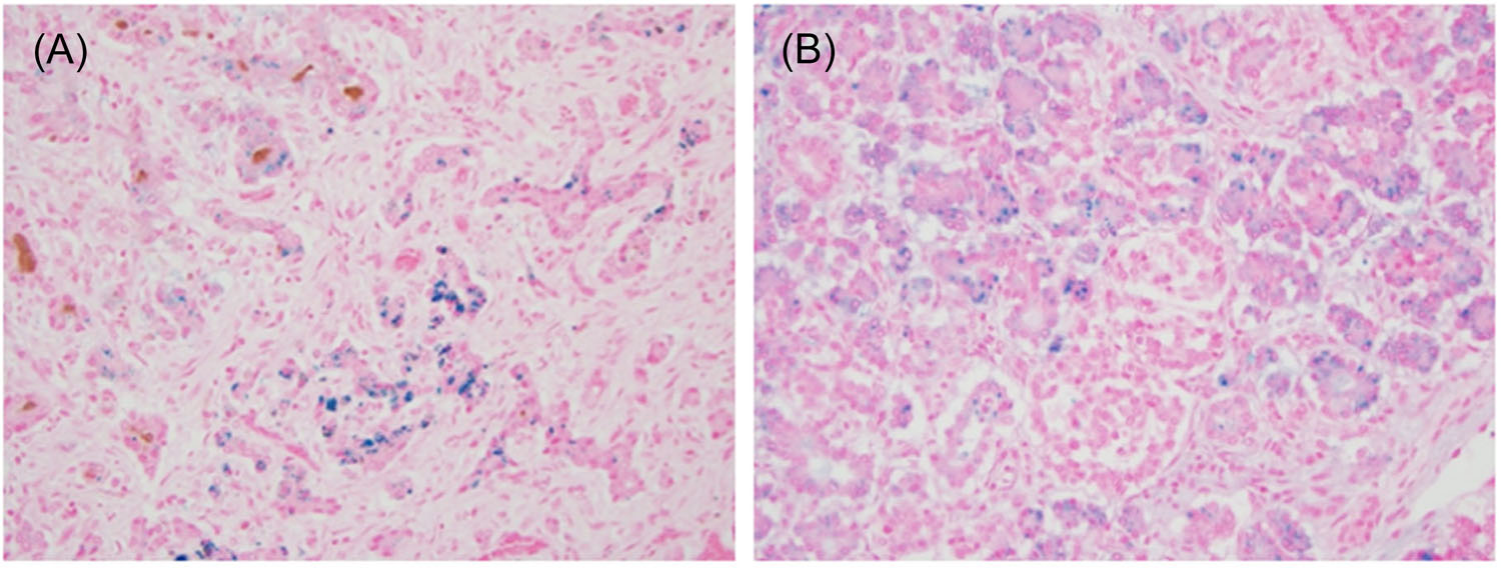
Iron stain shows increased deposition in the liver (A, ×400) and pancreas (B, ×400).

Post‐mortem analysis was notable for whole exome sequencing, which returned two variants in the leucyl‐tRNA synthetase 1 gene (*LARS1*), representing infantile liver failure syndrome type 1 (ILFS1), a maternally inherited deletion [chr5:146178476‐146186311] classified as pathogenic and a paternally inherited variant of uncertain significance (c.2244 C>G [p.Asp748Glu]). At autopsy, the liver was grossly shrunken and nodular. Microscopically, it showed severe parenchymal collapse with pseudoacinar and giant cell transformation, bile plugs, fatty infiltration, and numerous regenerative nodules with severe background fibrosis, suggesting hepatic failure with chronic changes (Figure 1). Increased iron deposition was highlighted by iron stain in the liver, pancreatic acinar epithelium, thyroid follicles, heart, airway submucous glands (focal faint), and renal tubules (focal faint) (Figure 2). The spleen was negative for reticuloendothelial staining, and the bone marrow showed mild staining. The morphological changes in the liver with increased iron deposition in the hepatocytes in addition to the extrahepatic siderosis are all features described in GALD. Placental pathology was notable for severe placental insufficiency, maternal vascular malperfusion, and massive perivillous fibrinoid deposition (MPFD). Both the placental MPFD and GALD‐related liver pathology are associated with a maternal‐fetal alloimmune mechanism, which raised the initial question of co‐occurrence of ILFS1 and GALD.

The autopsy demonstrated severe cholestatic liver disease with parenchymal collapse, regenerative nodules, fibrosis, and increased iron deposition with extrahepatic siderosis consistent with neonatal hemochromatosis (NH), representing the first case to date of confirmed ILFS1 with histopathological evidence of NH.

## 3 DISCUSSION

The histopathology of ILFS1 is rarely reported in the literature.^1^, ^2^, ^3^, ^4^ This is the first reported case of a NH phenotype in the setting of ILFS1. The severity of this case is atypical for ILFS1. Although extremely rare,^1^ ILFS1 is typically diagnosed in the first years of life with failure to thrive, developmental delay, encephalopathy, anemia, and chronic liver dysfunction with recurrent exacerbations following childhood illnesses.^2^ Two other case reports demonstrated severe manifestations in the neonatal period.^2^, ^4^

Liver histologic findings reported in ILFS1 may be variable, including steatosis, cirrhosis, and fibrosis. One of the reported severely affected neonatal patients showed fulminant hepatitis‐like hepatocellular injury, and fibrogenesis like our case.^4^ However, the extrahepatic siderosis on autopsy in our case is a novel finding that has not previously been published in the ILFS1 literature.

While extrahepatic iron deposition is a key feature of GALD, it has been demonstrated in other causes of neonatal liver disease, including non‐GALD cases of neonatal acute liver failure (ALF).^5^ Based on histologic differences from GALD and an identified genetic etiology for neonatal ALF, the most accurate assessment would be that this case represents the first reported case of ILFS1 with a NH phenotype.

## 4 CONCLUSION

Our case presented a diagnostic challenge in that the infant had other, more common reasons for her initial presentation of abdominal distension and coagulopathy. The differential for neonatal ALF is broad (sepsis, disseminated intravascular coagulopathy, GALD, infectious hepatitis, metabolic disorders, hemophagocytic lymphohistiocytosis). Whole‐exome sequencing should be considered in neonates with unexplained early liver failure. Lastly, autopsy and placental pathology are invaluable to add to the body of literature to more accurately differentiate between histological markers of rare diseases.

## CONFLICT OF INTEREST STATEMENT

The authors declare no conflicts of interest.

## ETHICS STATEMENT

Informed parental consent was obtained for publication of the case details.
